# Neuropsychological outcomes of children with Optic Pathway Glioma

**DOI:** 10.1038/s41598-020-59896-2

**Published:** 2020-02-24

**Authors:** Chiara Papini, Robert A. Dineen, David A. Walker, Shery Thomas, Nicola J. Pitchford

**Affiliations:** 1 School of Psychology, University of Nottingham, Nottingham, UK; 2 Children’s Brain Tumour Research Centre, University of Nottingham, Nottingham, UK; 3 Division of Clinical Neuroscience, School of Medicine, University of Nottingham, Nottingham, UK; 4 Sir Peter Mansfield Imaging Centre, School of Medicine, University of Nottingham, Nottingham, UK; 5 NIHR Nottingham Biomedical Research Centre, Queen’s Medical Centre, University of Nottingham, Nottingham, UK; 6 Division of Child Health, Obstetrics and Gynaecology, School of Medicine, University of Nottingham, Nottingham, UK; 7 Ophthalmology Department, Nottingham University Hospitals, Nottingham, UK

**Keywords:** CNS cancer, Paediatric cancer

## Abstract

Optic Pathway Glioma (OPG) is a relatively common brain tumour in childhood; however, there is scarce understanding of neuropsychological sequelae in these survivors. In this study, 12 children with diagnosis of OPG before 6 years of age received a comprehensive standardised assessment of visual perception, general intelligence and academic achievement, using adjustments to visual materials of the tests, to examine the extent of concurrent impairment in these functional domains. Information about vision, clinical and socio-demographic factors were extracted from medical records to assess the associations of neuropsychological outcomes with clinical and socio-demographic factors. Children with OPG exhibited high within-patient variability and moderate group-level impairment compared to test norms. Visual perception was the most impaired domain, while scholastic progression was age-appropriate overall. For cognition, core verbal and visuo-spatial reasoning skills were intact, whereas deficits were found in working memory and processing speed. Visual function was associated with tasks that rely on visual input. Children with OPG are at moderate risk of neuropsychological impairment, especially for visual perception and cognitive proficiency. Future research should elucidate further the relative contribution of vision loss and neurofibromatosis type 1 co-diagnosis within a large sample.

## Introduction

Optic Pathway Gliomas (OPGs) represent about 5% of all intracranial neoplasms in childhood^1,2^. They are a relatively homogeneous group of benign lesions that arise in any structure along the visual pathway and histologically classify as pilocytic astrocytoma. Half or more OPGs are associated with Neurofibromatosis type 1 (NF1)^3,4^, a multisystem genetic disorder that is associated with neuropsychological sequelae, including mild intellectual impairment, inattention, visuospatial deficits and learning disability^5–9^. While survival rates of OPG exceed 90%^10^, most children experience some degree of visual impairment that significantly affects their quality of life^11,12^.

Both NF1 comorbidity and visual impairment place children with OPG at risk of neuropsychological difficulties, but research has rarely focused on functional outcomes beyond vision. Retrospective investigations of OPG survivorship at two large north American institutions showed that only 26% to 42% of the whole patients cohort received sufficient psychological evaluation, and they reported only broad measures of Intelligence Quotient (IQ)^13^, in a descriptive manner^10^. One prospective study^14^ documented the neuropsychological profile of 21 school-aged children with OPG initially treated with chemotherapy integrating an IQ test for cognitive function with a collection of subtests from different batteries for individual perceptual and scholastic skills. This study was limited by the fact that over a third of the children had been treated with additional radiotherapy, the most critical risk factor for neurocognitive decline^15,16^. In addition, this does not reflect the modern approach to OPG management, which involves chemotherapy alone for cases requiring treatment^17^. In line with this, another prospective study^18^ evaluated 8 children with OPG (with hypothalamic involvement) before and after chemotherapy, but outcome measures included only IQ and executive functions tested through a variety of batteries. Finally, within the NF1 literature, children with diagnosed or treated OPG have typically been excluded from neuropsychology studies^5,6,19^, whereas the very few studies that attempted to disentangle the relative contribution of NF1 and brain tumour did not always focus on OPG^20–22^.

Although small sample size is an inherent limitation of research within this relatively rare condition, drawing conclusions from the available literature is hindered by problems with the analyses. When conducted^10^, statistical analyses consisted of between- or within-group comparisons involving two or more conditions or timepoints, without adopting multiple testing corrections^14,18^. For example, Lacaze *et al*^14^. compared 21 school-aged children dividing them into three subgroups based on NF1 status and treatment type, whereas Riva *et al*^18^. compared IQ scores before and after chemotherapy in 8 children. Furthermore, comparisons were conducted at the level of single subtests, whose examination is typically recommended only for within-subject analyses and individual profile description;^23^ indeed, pinpointing to such specific impairments to characterise a clinical population is problematic with small samples. Conversely, group means of broader indices have been reported only in descriptive manner^10,14^, without taking advantage of the possibility offered by standardised assessments to compare group means with the test normative mean. Finally, a further limitation is that none of the above studies stated whether adaptations to standardised assessment were used to address the visual impairment possibly experienced by these patients, as recommended by the recent European guidelines for assessing quality of survival in childhood brain tumour trials^24^.

The current study advances on previous research by investigating long-term outcomes on several neuropsychological domains in a group of patients who are representative of an OPG cohort treated and assessed following recent protocols. Specifically, this study integrates the assessment of general intelligence with two additional domains, namely scholastic attainment and visual perception. First, research on childhood brain tumour suggests that working memory, attention span, and in particular processing speed are the most affected domains^25,26^ and significantly impact on scholastic development^27–30^. Which cognitive and academic skills are affected in children with OPG and how they are interrelated has not yet been investigated. Second, the domain of visual perception is highly relevant due not only to the nature of OPG, which affects the visual system, but also for NF1 comorbidity, that is characterised by visuo-perceptual deficits^6,31^ even with normal IQ^32^. For these reasons, investigation of visual perception in the OPG population requires a clear construct definition^33^ that has not been provided in previous research^14^. According to hierarchical models of visual processing, visual perception is the ability to process physical features of visual stimuli, hence it is the intermediate level between sensation (i.e., the automatic reaction of the receptor cells, for example visual acuity) and cognition (i.e., the elaboration of abstract and symbolic properties, for example reading)^34^. Studies on children with typical development and with cancer history suggest that visuoperceptual and visual-motor skills support the acquisition of cognitive and scholastic abilities, such as reading and maths^35–41^. While it is not possible to fully disentangle input and processing problems within the visual stream, concurrent examination of different levels of visual processing and their relationships will help to better characterise the type of impairment experienced by young OPG patients.

An additional aim of the present study was to determine if clinical and socio-demographic characteristics are associated with neuropsychological abilities among children with history of OPG. Fouladi and colleagues^13^ argued that the OPG itself is responsible for cognitive impairment, as they found similar cognitive impairment at diagnosis and decline over time in children with and without NF1; however another study showed that young patients with NF1 co-diagnosis underperformed on many cognitive indices compared to non-NF1 patients^14^. Based on the literature of childhood brain tumour, younger age at diagnosis is associated with worst cognitive outcomes^13,42,43^ but adaptive compensatory mechanisms of superior verbal-auditory skills might also occur in vision-related tumours^44^. Finally, high socio-economic status (SES) is a protective factor for intellectual and scholastic^45^ development, whereas evidence is mixed regarding outcome status across male^42^ or female^43^ patients, which may be further influenced by the NF1 comorbidity^46^. To date, these factors have only been partially investigated in children with OPG^14^. While OPG can be considered relatively favourable tumours, identifying factors that increase risk of neuropsychological sequalae will enable more targeted intervention to be developed.

Overall, this study reports the development of visuo-perceptual, intellectual and scholastic abilities in 12 children with OPG (with and without NF1) managed with chemotherapy or observation. The objectives were to explore: (1) the extent of impairment in visual perception, intellectual function and scholastic attainment, (2) the associations across these three domains and (3) the relationships with clinical and socio-demographic factors.

## Methods

### Participants

This cross-sectional study was part of a project investigating neuroimaging predictors of cognitive outcomes in young brain cancer survivors. Potential participants were identified through NHS registers of children treated or referred to the Queen’s Medical Centre (Nottingham UK) between 2006 and 2017. Only children with diagnosis of tumour in the optic pathway are reported in this study. Additional inclusion criteria were: (i) age between 6 and 16 years, (ii) off-treatment for at least 6 months. Ethical approval was granted from the NHS Camberwell St Giles Research Ethics Committee and all procedures were performed in accordance with the Helsinki declaration. Written informed parental consent and child assent were obtained for each participant prior to participation.

### Vision examination

Children underwent a routine ophthalmologic examination at a tertiary referral paediatric neurosciences center. Visual acuity (VA) was measured through an age- and literacy-appropriate test, either the Bailey-Lovie test^47^, Crowded Kay Picture test^48^ or Keeler LogMAR Crowded acuity test^49^. VA scores of each eye were reported in logMAR scale and VA scores of each pair of eyes were classified using the criteria of the 2014 SIOP-e NF1 OPG Nottingham Workshop^50^.

### Neuropsychological assessment

Neuropsychological outcomes were assessed through standardised tests used widely in clinical and research work. Before study commencement, each subtest was reviewed to determine if it required vision (i.e. it cannot be completed with eyes closed), fine motor response (i.e. it requires the child to draw or write) and timed performance (i.e. the child is asked to perform as quickly as possible). See Supplementary Table S1.

Visuo-perceptual skills were assessed using the Developmental Test of Visual Perception, either 2^nd^ edition (DTVP-II;^51^ for ages 4–10 years) or Adolescent and Adult edition (DTVP-A;^52^ for ages 11–74 years). These tests consist of 8 and 6 subtests respectively and provide two analogous indices: Motor-Reduced Perception and Visual-Motor Integration. Tasks of both indices rely on vision, but only Visual-Motor Integration requires fine motor skills mainly with untimed performance. A composite index of General Vision Perception was obtained.

Cognitive abilities were evaluated with the Wechsler Intelligence Scale for Children, 4^th^ UK edition (WISC-IV^UK^^53^; for ages 6–16 years). It consists of 10 core subtests that provide four indices of broader abilities: Verbal Comprehension, Perceptual Reasoning, Working Memory and Processing Speed. Verbal Comprehension and Working Memory involve verbal-auditory input and output; Perceptual Reasoning and Processing Speed rely on vision, with Processing Speed requiring fine motor skills and timed performance. Two composite indices were obtained: Full-Scale IQ which is based on all four individual indices, and General Ability index which is based on Verbal Comprehension and Perceptual Reasoning.

Scholastic attainment was assessed using the Wechsler Individual Achievement Test, 2^nd^ UK edition (WIAT-II^UK^^54^; for ages 4–16 years). It consists of 10 core subtests that provide four indices of broader academic abilities: Reading, Mathematics, Written Language and Oral Language. All indices rely on vision, but only Mathematics and Written Language require fine motor response. A Total Composite index was obtained.

The neuropsychological assessment was conducted in a quiet area at the child’s home over one to three sessions, within four consecutive weeks. All children wore their glasses, if prescribed. For subtests that required vision, children with moderate or severe visual impairment were allowed to hold print material close for tasks that required a fine motor response (regardless of the timing) and to use their own magnifier device for motor-free subtests. No adaptation was used with children with monocular/binocular normal vision or mild visual impairment (best corrected VA ≥ 0.50 logMAR score) to minimise disruption of standardised procedures.

Neuropsychological performance was scored following the test manuals and raw scores were transformed into standard scores (μ = 100, σ = 15) for each index. Scores were classified as: clinical impairment (below -2 SD), deficit (−1 to −2 SD), average (−1 to +1 SD), above-average (+1 to +2 SD) and gifted (above +2 SD). Socio-economic status (SES) was estimated through postcodes using the Income Deprivation Affecting Children Index rank 2015^55^. Values range from 1 to 32,482, with lower scores indicating more disadvantaged areas.

### Statistical analyses

Analyses were conducted in R (version 3.5.0)^56^ through R Studio (version 1.1.456)^57^. Sample biases were assessed comparing eligible patients who did and did not participate in terms of age, sex and NF1 comorbidity through independent-sample t-tests and chi-square tests. Chi-square tests with Fisher’s exact correction were used to assess differences between sporadic and syndromic OPGs on socio-demographic and clinical characteristics. All individual and composite indices were analysed using one-sample *t*-tests to assess deviations from the test norms. For comparison with previous research^14^, at the cognitive level, paired-sample and independent-sample t-tests were conducted with Verbal Comprehension, Perceptual Reasoning and Full-Scale IQ to investigate discrepancies across indices and differences between sporadic and syndromic cases for these measures. Confidence intervals at 95% level were computed and effect sizes were estimated as Cohen’s *d*^58^.

Pearson correlations were conducted to examine the associations across the three domains. Finally, several socio-demographic and clinical factors in relation to neuropsychological outcomes were considered. Associations with sex (1: female, 2: male), tumour type (1: sporadic, 2: syndromic) and visual function (1: poor, requiring visual aids, 2: good, not requiring visual aids) were examined using point-biserial correlations. The relationship with visual acuity (VA in the best eye) and SES was evaluated using Spearman’s *ρ* correlations. Pearson’s *r* correlations were conducted to examine the impact of age at diagnosis and time post treatment on OPG outcomes. All correlation analyses were conducted on composite indices and Bonferroni correction was applied (α = 0.05/4 = 0.0125). Further correlations using individual indices were explored with the same alpha level of 0.0125.

## Results

### Sample characteristics

Of 27 eligible patients with OPG, 12 agreed to take part. Participating and non-participating children did not differ in age at diagnosis (*t* = 1.08, *P* = 0.292), sex (χ^2^ = 1.50, *P* = 0.398) and NF1 co-diagnosis (χ^2^ = 0.49, *P* = 0.683). There were 7 boys and 5 girls; 7 children (4 boys) had co-diagnosis of NF1. SES ranged from 8,498 to 32,309 (*Mdn* = 25,242.5). Mean age at assessment was 10.1 years (*SD* = 2.2; range: 6.2 – 13.7); all were diagnosed with OPG before 6 years of age (*M (SD)* = 2.5 (1.6); range: 0.7 – 5.6). Three children did not receive treatment for OPG; the others received chemotherapy. There were no significant differences between sporadic and NF1-associated OPGs in terms of sex (χ^2^ = 0.01, *P* = 1.00) and treatment (χ^2^ = 0.11, *P* = 1.00).

### Visual function

**Figure 1:**
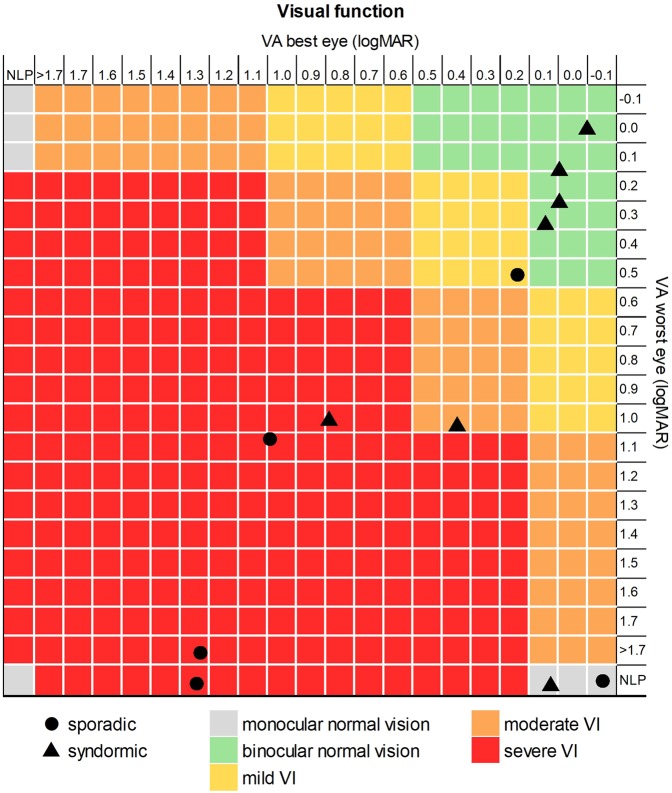
Classification of the visual acuity scores of the best (x-axis) and worst (y-axis) eyes of each study participant, either with sporadic (circles) or syndromic (triangles) OPG. Children were grouped into five categories: binocular normal vision (green), monocular normal vision (grey), mild visual impairment (yellow), moderate visual impairment (orange) and severe visual impairment (red). VA = visual acuity; VI = visual impairment; NLP = no light perception.

Figure 1 portrays visual function of the participants. Five children had visual impairment (moderate 1/12, 8%; severe 4/12, 33%) and used low vision aids during the neuropsychological assessments. Among the other children, 1/12 (8%) had mild visual impairment, 4/12 (33%) had binocular normal vision, and 2/12 (17%) had normal vision in the best eye with no perception of light in the worst eye. There were no differences in visual aid use between sporadic and NF1 cases (χ^2^ = 1.19, *P* = 0.558).

### Neuropsychological outcomes: extent of impairment

**Figure 2:**
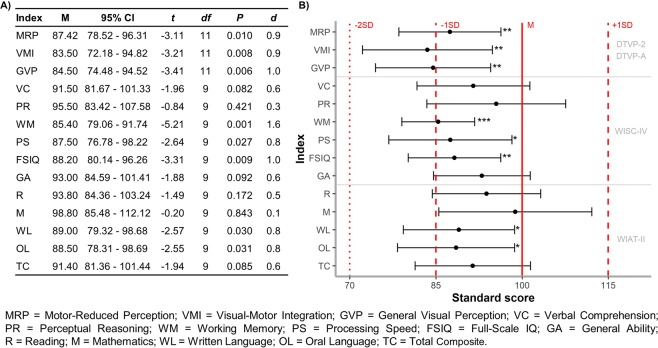
**(A)** Descriptive statistics (*M* and 95% *CI*) and results of the one-sample t-tests (*t* statistic, *P* values and Cohen’s *d*). **(B)** Mean (dot) and 95% confidence interval (error bar) of each test (individual and composite indices). Vertical red lines represent test norms: mean (solid), 1 standard deviation (dashed) and 2 standard deviation (dotted). *P ≤ 0.05, **P ≤ 0.01, ***P ≤ 0.001.

Two children did not complete the cognitive and scholastic tests due to fatigue at sustaining multiple sessions. Table 1 summarises individual and group results for the neuropsychological assessments. Figure 2 reports group means and comparisons to test norms.

**Table 1:** Individual profile of neuropsychological performance of the study participants on individual and composite indices; group statistics (mean and standard deviation) compared to test norms given below.

Case	NF1	Visual function	Aid	Visual perception			Cognitive function						Scholastic attainment				
				MRP	VMI	GVP	VC	PR	WM	PS	FSIQ	GA	R	M	WL	OL	TC
P1	Yes	Normal	No	A	A	A	A	A	–	A	A	A	A	A	−	A	−
P2	Yes	M – Normal	No	— —	— —	— —	A	A	–	A	A	A	A	+	A	A	A
P3	Yes	Normal	No	A	A	A	A	A	A	A	A	A	A	A	A	A	A
P4	Yes	Moderate VI	Yes	−	— —	— —	A	— —	−	— —	— —	−	—	— —	— —	−	— —
P5	Yes	Normal	No	−	— —	−	n. t.	n. t.	n. t.	n. t.	n. t.	n. t.	n. t.	n. t.	n. t.	n. t.	n. t.
P6	Yes	Normal	No	A	A	A	n. t.	n. t.	n. t.	n. t.	n. t.	n. t.	n. t.	n. t.	n. t.	n. t.	n. t.
P7	Yes	Severe VI	Yes	— —	−	−	— —	A	−	−	−	−	−	−	−	— —	−
P8	No	M – Normal	No	A	A	A	−	+	A	A	A	A	A	A	A	−	A
P9	No	Mild VI	No	A	A	A	A	A	A	A	A	A	A	A	A	A	A
P10	No	Severe VI	Yes	−	— —	— —	+	A	A	A	A	A	A	+ +	A	A	+
P11	No	Severe VI	Yes	−	— —	— —	A	A	A	— —	A	A	A	A	−	A	A
P12	No	Severe VI	Yes	A	A	A	A	−	A	A	−	−	A	A	A	−	A
*M*				87.4*	83.5*	84.5*	91.5	95.5	85.4*	87.5*	88.2*	93.0	93.8	98.8	89.0*	88.5*	91.4
*SD*				14.0	17.8	15.8	13.7	16.9	8.9	15.0	11.3	11.8	13.2	18.6	13.5	14.2	14.0

MRP = Motor-Reduced Perception; VMI = Visual-Motor Integration; GVP = General Visual Perception; VC = Verbal Comprehension; PR = Perceptual Reasoning; WM = Working Memory; PS = Processing Speed; FSIQ = Full-Scale IQ; GA = General Ability; R = Reading; M = Mathematics; WL = Written Language; OL = Oral Language; TC = Total Composite.

Visual function: VI = Visual Impairment, M = Monocular vision.

Scores: “A” = between + 1 and −1 SD; “−” = between −1 and −2 SD.; “— —” = below −2SD.; “+” = between + 1 and + 2 SD; “++” = above + 2 SD; “n. t.” = not tested.

*P < 0.05 at least.

For visual perception, a significant difference from test norms was found for both individual indices Motor-Reduced Perception and Visual-Motor Integration, and for the composite index General Visual Perception.

For intellectual function, there was a significant difference from test norms for Full-Scale IQ, but not General Ability. Scores for Working Memory and Processing Speed were significantly depressed compared to test norms. No significant differences were found between Verbal Comprehension and Perceptual Reasoning (*t*_9_ = 0.58, *P* = 0.577). No significant differences were found between syndromic and sporadic cases for either Full-Scale IQ (82.6 VS 93.8; *P* = 0.120), Verbal Comprehension (88.6 VS 94.4; *P* = 0.537), or Perceptual Reasoning (89.0 VS. 102.0; *P* = 0.245).

For scholastic achievement, a deviation from test norms was found for two abilities, Written Language and Oral Language, but not the Total Composite index.

### Neuropsychological outcomes: associations across domains

Table 2 shows associations between all the neuropsychological indices. Across domains, strong correlations for composite indices were found between scholastic attainment and cognitive functioning (both Full-Scale IQ and General Ability; *r* ≥ 0.85, *P* ≤ 0.002), whereas no significant correlations were found for visual perception. On individual indices, one medium-to-strong correlation was found between the perception index of Visual-Motor Integration and the visuo-spatial cognitive index of Processing Speed (*r* = 0.68, *P* = 0.031), but none between perception and academic attainment. Several moderate-to-strong correlations were found between individual indices of cognitive and scholastic abilities (*r* ≥ 0.65, *P ≤ *0.043).

**Table 2:** Pearson’s *r* coefficients for the correlations between all the neuropsychological measures.

	Visual perception			Cognitive functioning						Scholastic attainment				
	MRP	VMI	GVP	VC	PR	WM	PS	FSIQ	GA	R	M	WL	OL	TC
MRP	—													
VMI	0.72**	—												
GVP	0.91***	0.94***	—											
VC	0.14	−0.38	−0.16	—										
PR	0.58	0.38	0.50	−0.01	—									
WM	0.62	0.38	0.52	0.39	0.15	—								
PS	0.53	0.68*	0.65*	−0.08	0.74*	0.32	—							
FSIQ	0.65*	0.30	0.48	0.53	0.76*	0.57	0.73*	—						
GA	0.53	0.02	0.26	0.68*	0.72*	0.39	0.49	0.93***	—					
R	0.56	0.22	0.38	0.56	0.60	0.71*	0.65*	0.94***	0.84***					
M	−0.05	−0.22	−0.17	0.54	0.41	0.29	0.51	0.70*	0.68*	0.77**	—			
WL	0.54	0.58	0.59	0.18	0.61	0.55	0.92***	0.81**	0.58	0.80**	0.60	—		
OL	0.27	−0.14	0.03	0.84**	0.42	0.35	0.34	0.79**	0.89***	0.75*	0.73*	0.54	—	
TC	0.37	0.10	0.22	0.63	0.55	0.56	0.66*	0.92***	0.85**	0.96***	0.89***	0.81**	0.85**	—

MRP = Motor-Reduced Perception; VMI = Visual-Motor Integration; GVP = General Visual Perception; VC = Verbal Comprehension; PR = Perceptual Reasoning; WM = Working Memory; PS = Processing Speed; FSIQ = Full-Scale IQ; GA = General Ability; R = Reading; M = Mathematics; WL = Written Language; OL = Oral Language; TC = Total Composite.

Significance at uncorrected *p* values: *P ≤ 0.05, **P ≤ 0.01, ***P ≤ 0.001. Bold indicates correlations that survived Bonferroni correction (α = 0.05/4 = 0.0125).

### Impact of clinical and socio-demographic factors

Table 3 shows the relationship of neuropsychological outcomes with clinical and socio-demographic factors. Visual function was significantly associated with Perceptual Reasoning, Processing Speed and Written Language (all *P* < 0.05), demonstrating worse performance in children with poor vision requiring low vision aids compared to those with sufficiently intact vision (although these did not survive Bonferroni correction). The other clinical and socio-demographic factors did not correlate significantly with neuropsychological outcomes. The direction of the effects was mixed for all variables except for the NF1 status, indicating that children with sporadic OPG consistently obtained lower scores on all indices (Supplementary Table S2).

**Table 3:** Correlations (point-biserial coefficient rpb, Spearman’s ρ, Pearson’s r) across the neuropsychological indices of visual perception, cognitive function and scholastic attainment in relation to clinical and socio-demographic factors.

Domain	Index	Tumour type (*r*_*pb*_)	Visual function (*r*_*pb*_)	VA score (*ρ*)	Age at diagnosis (*r*)	Time after treatment (*r*)	Sex (*r*_*pb*_)	Socio-economic status (*ρ*)
Visual perception	MRP	−0.36	0.52	−0.47	0.37	−0.05	−0.06	−0.18
	VMI	−0.07	0.50	−0.47	0.31	−0.49	−0.40	−0.07
	GVP	−0.21	0.54	−0.48	0.36	−0.34	−0.27	−0.22
Cognitive functioning	VC	−0.22	−0.02	0.17	< −0.01	0.58	0.25	0.45
	PR	−0.41	0.64*	−0.46	0.38	0.25	0.57	0.18
	WM	−0.55	0.07	0.15	−0.26	0.55	−0.33	0.12
	PS	−0.29	0.67*	−0.63*	0.22	0.04	0.08	0.23
	FSIQ	−0.52	0.52	−0.40	0.19	0.56	0.36	0.36
	GA	−0.47	0.45	−0.23	0.28	0.60	0.59	0.22
Scholastic attainment	R	−0.64*	0.29	−0.10	−0.03	0.61	0.27	0.13
	M	−0.44	0.07	0.15	−0.25	0.71	0.43	0.54
	WL	−0.26	0.64*	−0.61	0.20	0.18	0.03	0.29
	OL	−0.23	0.39	−0.27	0.27	0.63	0.53	0.61
	TC	−0.50	0.33	−0.21	< 0.01	0.63	0.35	0.37

Note: tumour type (1: sporadic, 2: syndromic), visual function (1: poor, requiring visual aids, 2: good, not requiring visual aids) and sex (1: female, 2: male).

VA = visual acuity; MRP = Motor-Reduced Perception; VMI = Visual-Motor Integration; GVP = General Visual Perception; VC = Verbal Comprehension; PR = Perceptual Reasoning; WM = Working Memory; PS = Processing Speed; FSIQ = Full-Scale IQ; GA = General Ability; R = Reading; M = Mathematics; WL = Written Language; OL = Oral Language; TC = Total Composite.

*Significant correlations at uncorrected *P* ≤ 0.05; none survived at corrected α = 0.0125.

## Discussion

This is the first study documenting low-level visual acuity, high-level visual perception, verbal and visuo-spatial intellectual function and real-life scholastic abilities in children with OPG managed with observation or chemotherapy.

Analyses of individual profiles showed visual perception was the most affected area, with half of the sample underperforming. For intellectual function and academic attainment, most children performed on average for each domain. Neuropsychological outcomes displayed a high degree of heterogeneity and complex relationships with visual impairment and NF1 co-diagnosis. Of note, three children (two sporadic cases) performed at or above average for some indices, whereas two children with moderate or severe visual impairment and co-diagnosis of NF1 (treated with chemotherapy) performed below average on almost all abilities. This result corroborates findings from a previous case series describing cognitive deterioration after chemotherapy only in 3 children with NF1-associated OPG, who also had very poor vision at diagnosis^18^. At the group level, none of the indices were clinically impaired (≤ −2 SD), but all mean scores were in the lower range of average performance or just below −1SD. The underperformance in comparison to the test norm μ = 100 was statistically significant for more than half of the indices (2/4 composite and 6/10 individual indices).

Visual perception was found to be a marked area of weakness in paediatric OPG patients as group performance of all measures was significantly below the test mean. Difficulties with processing visual-perceptual stimuli, even without motoric response and after low vision adjustment, raise questions about the reliability of visual acuity tests in these patients^59^, when children are required to analyse the physical properties of a stimulus to recognise and name a shape or letter without meaningful context. Visual-perceptual processing and visual-motor integration were altered in children with low vision^60^, and weaknesses in these areas are also well established among NF1 children^6,7,31^. Our small sample did not afford the relative contribution of visual function and NF1 status to be elucidated. However, these results indicate that visual perception should be incorporated into clinical assessments of children with OPG.

Amongst composite indices of intellectual function, the mean score of General Ability did not differ from test norms, whereas the mean score of Full-Scale IQ was significantly below. General Ability comprises only of Verbal Comprehension and Perceptual Reasoning, both of which were preserved. In contrast, Full-Scale IQ also includes Working Memory and Processing Speed, which showed significant weaknesses in our sample. Therefore, the discrepancy between average General Ability and statistically-reduced Full-Scale IQ demonstrates the weaknesses in Working Memory and Processing Speed, which reflects the proficiency and efficiency of cognitive processing^61^. A similar pattern of results was found in a heterogeneous group of young brain tumour survivors^62^, where all children received radiation therapy and all cognitive measures were below the test mean. Our study provides promising results for non-irradiated OPG patients as it demonstrates that core verbal and visuo-spatial reasoning abilities remain intact in these children. Nonetheless, the deficit shown on general intelligence when Working Memory and Processing Speed are considered indicates that these two cognitive domains require clinical attention as they are critical for intellectual development^63–65^ and scholastic progression^66,67^.

At the cognitive level, there was no discrepancy between Verbal Comprehension and Perceptual Reasoning, and both mean scores were close to the test norm. Previous research by Lacaze and colleagues^14^ reported a significant difference between intact verbal IQ and depressed performance IQ (analogous indices in the WISC-III), but their results were confounded by the presence of irradiated children in their sample and the lack of control over visual input during neuropsychological assessment. On the contrary, our study with non-irradiated OPG patients demonstrated similar development of Verbal Comprehension and Perceptual Reasoning when children use visual magnifiers. This ecological approach, which is in line with the recommendations of the Brain Tumour Quality of Survival Group, International Society of Paediatric Oncology (Europe)^24^, showed that when visual stimuli are made accessible, visuo-perceptual reasoning in children with OPG develops adequately for the child’s age and is similar to their verbal comprehension skills. In addition, compared to Lacaze and colleagues^14^, our study showed no differences between sporadic and syndromic cases not only on verbal IQ measure, but on visual-spatial and total IQ scores. Although these novel results could arise from the adaptation of visual input, subgroup mean scores support a tendency towards better performance in children with sporadic OPGs compared to those with NF1 co-diagnosis. Of note, these analyses were conducted only for replication purpose, systematic investigation with larger samples is warranted in future.

Our results revealed relatively preserved scholastic attainment compared to other domains, with some children performing above average on some measures. Children with OPG had appropriate scholastic attainment based on the Total Composite index and this was also confirmed on the individual indices of Reading and Mathematics. Mild impairment was found only in Written Language and Oral Language. Underperformance on Written Language might be attributable to visual-motor and handwriting difficulties, but also spelling deficits that can result from either low vision^68,69^ or NF1 syndrome^70,71^. The impairment in Oral Language might reflect difficulties at processing fine details of visual stimuli necessary for many of these tasks.

Across domains, significant associations between composite indices of visual perception and cognitive and scholastic abilities were not found. However, some moderate-to-strong correlations emerged for individual indices across domains that rely on vision and/or fine motor response, although these did not reach statistical significance (for example, between Motor-Reduced Perception and Perceptual Reasoning and between Visual-Motor Integration and Written Language), probably because of the small sample size. Therefore, associations/dissociations across these three domains should be explored further in larger studies before excluding the potential utility of visual rehabilitation programs for these patients.

Finally, among the clinical and socio-demographic factors, visual function showed medium-to-strong positive correlations with some cognitive and scholastic individual indices that were assessed with visual tasks. Consistently, VA in the best eye was negatively correlated with Processing Speed. This demonstrates that, even if children with poor vision were allowed low vision aids, their performance was significantly poorer in comparison to the children with relatively intact vision. It has been proposed that early visual loss may either hamper neuropsychological acquisition causing long-term detrimental effects, or facilitate timely neural re-organisation and behavioural adaptation resulting in adequate development^12^. Our results support the first proposition as children with poor visual function obtained lower scores on neuropsychological tests despite the use of low visual aids. This is consistent with results in adulthood reporting poor neurocognitive outcomes in low-grade glioma survivors with bilateral blindness compared to those without visual impairment^12^. Notably, NF1 demonstrated a consistent effect on all neuropsychological abilities, with syndromic cases underperforming in comparison to sporadic cases, although this was statistically significant only for Reading. This is consistent with findings by Lacaze and colleagues^14^, who also compared children with and without NF1 treated with front-line chemotherapy. While NF1 research tends to exclude patients with OPG due to brain abnormality or visual impairment, the results of this study suggests that overall deficits associated with OPG might be driven by NF1 co-diagnosis and therefore these children require more research and clinical attention, although there is a desperate need for reliable and standardised tools to evaluate visually impaired children^72^.

Strengths of this study include utilisation of comprehensive standardised tests (with high internal consistency across individual subtests) for each domain, the ecological use of low vision aids to address vision input at least in motor-free tasks, and the exclusion of neurotoxic effects due to radiation. The main limitation is low statistical power due to the small sample size, which increases the risk of false negative results. This was also a limitation of the only other previous report of neuropsychological sequelae of OPG which has additional critical confounders^14^. Our findings suggest that assessment of visual-perceptual processing, a marked weakness of OPG patients, should be included during routine ophthalmologic examinations. Future research could investigate differences between OPG patients with and without NF1 using a larger sample, and control for adaptations to visual materials, to elucidate the impact of vision loss on neuropsychological outcomes beyond information input.

## Supplementary information

Supplementary information.

## Data Availability

The datasets analysed during the current study are available from the corresponding author on reasonable request.
