# Supplementary material for: Neuropsychological outcomes of children with Optic Pathway Glioma
Source: Sci Rep. 2020 Feb 24;10:3344. doi: 10.1038/s41598-020-59896-2 (PMC7039908; doi:10.1038/s41598-020-59896-2)
Supplement: Supplementary file 1 — Supplementary information. [file 41598_2020_59896_MOESM1_ESM.pdf]

SUPPLEMENTARY MATERIALS

Neuropsychological outcomes of children with Optic Pathway Glioma

Chiara Papini, Robert A. Dineen, David A. Walker, Shery Thomas, Nicola J. Pitchford.

Table S1. Analysis of the characteristics of each neuropsychological subtest.

Domain	Test	Scale	Subtest	Vision	Fine motor	Timed
Visual perception	DTVP-2 / DVTP-A	MRP	Position in space [a]	1	0	0
			Figure-ground	1	0	0
			Visual closure	1	0	0
			Form constancy	1	0	0
		VMI	Eye-hand coordination [a]	1	1	0
			Copying	1	1	0
			Spatial relations [a]	1	1	0
			Visual-motor speed	1	1	1
			Visual-motor search [b]	1	1	1
Cognitive function	WISC-IV^{UK}	VC	Similarities	0	0	0
			Vocabulary	0	0	0
			Comprehension	0	0	0
		PR	Block design [c]	1	1	0/1
			Picture concept	1	0	0
			Matrix reasoning	1	0	0
		WM	Digit span	0	0	0
			Letter-number sequence	0	0	0
		PS	Coding	1	1	1
			Symbol searching	1	1	1
Scholastic attainment	WIAT-II^{UK}	R	Word reading	1	0	0
			Reading comprehension	1	0	0
			Pseudoword decoding	1	0	0
		M	Numerical operations	1	1	0
			Mathematica reasoning	1	0	0
		WL	Spelling	1	1	0
			Written expression	1	1	0
		OL	Listening comprehension	1	0	0
			Oral expression	1	0	1

MRP = Motor-Reduced Perception; VMI = Visual-Motor Integration; VC = Verbal Comprehension; PR = Perceptual Reasoning; WM = Working Memory; PS = Processing Speed; R = Reading; M = Mathematics; WL = Written Language; OL = Oral Language.

[a] Subtest included only in the DTVP-2.

[b] Subtest included only in the DTVP-A.

[c] In our sample, there was no discrepancy between scores with and without time bonus.

Table S2. Descriptive statistics (M and SD) and comparison to test norms (*t*-statistic and *P*-value) on each neuropsychological index for sporadic and syndromic OPGs.

		Sporadic			Syndromic		
	Index	*M* (*SD*)	*n*	*t*, *P*	*M* (*SD*)	*n*	*t*, *P*
Visual perception (DTVP-2/DTVP-A)	MRP	93.2 (13.2)	5	-1.15, .315	83.3 (14.0)	7	-3.17, .019
	VMI	85.0 (23.2)	5	-1.45, .222	82.4 (14.8)	7	-3.13, .020
	GVP	88.2 (18.6)	5	-1.42, .230	81.9 (14.3)	7	-3.35, .015
Cognitive functioning (WISC-IV^{UK})	VC	94.4 (13.8)	5	-0.91, .415	88.6 (14.6)	5	-1.74, 157
	PR	102.0 (15.5)	5	0.29, .787	89.0 (17.2)	5	-1.43, 225
	WM	90.0 (3.7)	5	-5.98, .004	80.8 (10.5)	5	-4.10, .015
	PS	91.6 (14.6)	5	-1.29, .268	83.4 (15.8)	5	-2.35, .079
	FSIQ	93.8 (8.6)	5	-1.60, .184	82.6 (11.5)	5	-3.38, .028
	GA	98.2 (9.8)	5	-0.41, .703	87.8 (12.1)	5	-2.25, .087
Scholastic attainment (WIAT-II^{UK})	R	101.8 (8.0)	5	0.50, .641	85.8 (13.0)	5	-2.45, .071
	M	106.6 (17.0)	5	0.87, .435	91.0 (18.4)	5	-1.09, .335
	WL	92.4 (11.3)	5	-1.51, .206	85.6 (16.0)	5	-2.01, .114
	OL	91.6 (10.7)	5	-1.76, .154	85.4 (17.8)	5	-1.83, .141
	TC	98.0 (10.8)	5	-0.41, .700	84.8 (14.8)	5	-2.30, .083

MRP = Motor-Reduced Perception; VMI = Visual-Motor Integration; GVP = General Visual Perception; VC = Verbal Comprehension; PR = Perceptual Reasoning; WM = Working Memory; PS = Processing Speed; FSIQ = Full-Scale IQ; GA = General Ability; R = Reading; M = Mathematics; WL = Written Language; OL = Oral Language; TC = Total Composite.
